# Oral Glutamine Is Superior Than Oral Glucose to Promote Glycemia Recovery in Mice Submitted to Insulin-Induced Hypoglycemia

**DOI:** 10.1155/2013/841514

**Published:** 2013-08-24

**Authors:** Amanda Nunes Santiago, Vilma Aparecida Ferreira de Godoi-Gazola, Mariana Fachin Milani, Vanessa Cristina de Campos, Vanessa Rodrigues Vilela, Maria Montserrat Diaz Pedrosa, Roberto Barbosa Bazotte

**Affiliations:** ^1^Department of Pharmacology and Therapeutics, State University of Maringá, 87020-900 Maringá, PR, Brazil; ^2^Department of Physiological Sciences, State University of Maringá, 87020-900 Maringá, PR, Brazil

## Abstract

The effect of the oral administration of blood glucose precursors on glycemia recovery and liver glucose production in fasted mice subjected to insulin-induced hypoglycemia (IIH) was investigated. IIH was obtained with increasing doses (from 0.5 to 2.0 U*·*kg^−1^) of intraperitoneal regular insulin where glycemia was evaluated from 0 to 300 min after insulin injection. The dose of 1.0 U*·*kg^−1^ showed the best results, that is, a clear glycemia recovery phase without convulsions or deaths. Thus, this dose was used in all experiments. Afterwards, mice submitted to IIH received orally by gavage: saline (control group), glucose (100 mg*·*kg^−1^), glycerol (100 mg*·*kg^−1^), lactate (100 mg*·*kg^−1^), alanine (100 mg*·*kg^−1^), or glutamine (100 mg*·*kg^−1^). It was observed that glutamine was more effective in promoting glycemia recovery if compared with glucose, lactate, glycerol, or alanine. In agreement with these results, the best performance in terms of liver glucose production was obtained when glutamine was used as glucose precursors. These results open perspectives for clinical studies to investigate the impact of oral administration of gluconeogenic amino acids to promote glycemia recovery during hypoglycemia.

## 1. Introduction

Diabetes is a chronic disease that requires careful monitoring and control. Without proper management it can lead to chronic hyperglycemia associated with long-term complications that include nephropathy, neuropathy, retinopathy, and several other disorders. It must be emphasized that all these complications could be prevented by intensive insulin therapy. However, intensive insulin therapy, essential for type 1 diabetic patients and some of those with type 2, has insulin-induced hypoglycemia (IIH) as its major adverse effect [1, 2]. Moreover, IIH can trigger significant neurologic alterations, neuronal death, coma, and death [3, 4].

To better understand the mechanisms of IIH we developed an experimental model in which hypoglycemia was obtained by an intraperitoneal injection of a pharmacological dose of regular insulin in nondiabetic Wistar rats. This animal model that has been used by our research group since 1994 [5] is suitable to investigate the metabolic changes induced by IIH [6–12].

 Thus, by using this rat model we obtained considerable progress in the elucidation of the mechanisms of IIH. For example, despite the paradigm that insulin inhibits hepatic gluconeogenesis, our previous studies in rats suggest that the counterregulatory mechanisms can surpass the inhibitory effects of insulin on liver gluconeogenesis [7–12]. In agreement with our studies, other investigations demonstrated that the administration of gluconeogenic precursors such as alanine [13], lactate [14], and pyruvate [15] during IIH decreased the risk of hypoglycemia and/or promoted glycemia recovery.

 Considering that the Swiss mouse is a suitable animal model for studies of regulation of glycemia and liver metabolism [16–19], in this study we evaluated the effect of the oral administration of hepatic glucose precursors on the glycemia and liver glucose production in mice subjected to IIH.

## 2. Materials and Methods

### 2.1. General Experimental Procedures

#### 2.1.1. Chemicals

Alanine and glutamine were obtained from ICN Biochemicals (Costa Mesa, CA, USA). NAD, NADH, and lactate dehydrogenase were obtained from Sigma Chemical Co. (St. Louis, USA). Regular insulin (Novolin) was purchased from Novo Nordisk (Brazil). All other reagents were of the the best available grade (98–99.8% purity).

#### 2.1.2. Animals

Adult male Swiss mice (*Mus musculus*) weighting 20–30 g were used. The animals had free access to water and food (Nuvilab rodent chow) and were kept under constant temperature (23 ± 1°C) until the day before the experiment. At this day the food was removed at 5:00 p.m. All experiments were done after an overnight fasting (5:00 p.m–7:00 a.m), so at the moment of the experiments the animals were at a 14 hr fasting. 

All experiments were approved by the Committee of Ethics in Animal Experimentation (079-PRO 051.2011).

### 2.2. Determination of the Glycemic Curve

The animals were divided into six groups according to the dose (U·kg^−1^ body weight) of intraperitoneal (ip) regular insulin (Novolin) that was given: 0.0 vehicle (*n* = 4), 0.1 (*n* = 4), 0.5 (*n* = 4), 1.0 (*n* = 9), 1.5 (*n* = 4), and 2.0 (*n* = 4). Seven blood samples were collected from the tail of each animal 0, 30, 60, 120, 180, 240, and 300 min after the ip injection of insulin or vehicle. The glycemia (mg·dL^−1^) was determined with the aid of a home glucometer (Optium Xceed). 

### 2.3. Evaluation of Glycemia after Oral Administration of Gluconeogenic Precursors in Mice Subjected to IIH

After insulin administration (1.0 U·kg^−1^), the animals were divided into six groups: saline (*n* = 38, i.e., 3–7 mice for each time), glucose 100 mg·kg^−1^ (*n* = 37, i.e., 3–6 mice for each time), glycerol 100 mg·kg^−1^ (*n* = 21, i.e., 2-3 mice for each time), lactate 100 mg·kg^−1^ (*n* = 17, i.e., 2-3 mice for each time), alanine 100 mg·kg^−1^ (*n* = 21, i.e., 2-3 mice for each time), or glutamine 100 mg·kg^−1^ (*n* = 22, i.e., 2-3 mice for each time). The gluconeogenic precursors, as well as saline and glucose (as control groups), were given orally through gavage 15 min after the insulin injection. The glycemia was determined with a home glucometer (Optium Xceed) from the tail blood at 0, 15, 30, 60, 120, 180, 240, and 300 min after ip insulin injection. 

### 2.4. Liver Perfusion Experiments

In another set of the experiments, the livers were isolated and perfused *in situ*. For this purpose, the mice received ip saline or ip regular insulin (1.0 U·kg^−1^), respectively, and 180 min after insulin (IIH group) or saline (NORMO group) injection they were anesthetized (ketamine 60 mg·kg^−1^ and xylazine 12 mg·kg^−1^, ip). Hypoglycemia was confirmed by the glycemia from tail blood just before anesthesia.

The perfusion fluid, the Krebs-Henseleit buffer (KHB), pH 7.4, 37°C, and saturated with a 95% : 5% O_2_ : CO_2_ mixture, was introduced (4 mL·min^−1^·g^−1^ of liver) through a cannula inserted into the portal vein. The composition of the KHB buffer was 115 mM NaCl, 25 mM NaHCO_3_, 5.8 mM KCl, 1.2 mM Na_2_SO_4_, 1.18 mM MgCl_2_, 1.2 mM NaH_2_PO_4_, and 2.5 mM CaCl_2_. The gluconeogenic precursors glycerol (*n* = 26 for NORMO group and *n* = 29 for HII group, i.e., 3–5 and 3–6 mice, resp., for each concentration), lactate (*n* = 33 for NORMO group and *n* = 26 for HII group, i.e., 3–8 and 3–6 mice, resp., for each concentration), alanine (*n* = 32 for NORMO group and *n* = 37 for HII group i.e., 3–7 and 3–8 mice, resp., for each concentration), or glutamine (*n* = 25 for NORMO group and *n* = 35 for HII group, i.e., and 3–6 mice, resp., for each concentration) were dissolved in the perfusion fluid at increasing concentrations of 0.5, 1.0, 2.0, 4.0, 8.0, 12.0, and 16.0 mM. The effluent perfusate from the liver was collected at intervals of 5 min through a cannula inserted into the inferior vena cava. At the end of the experiment, the liver was removed and weighted so that the liver production of glucose, lactate [20], pyruvate [21], and urea [22] can be expressed by gram of liver (*μ*mol·min^−1^·g^−1^). 

**Figure 1:**
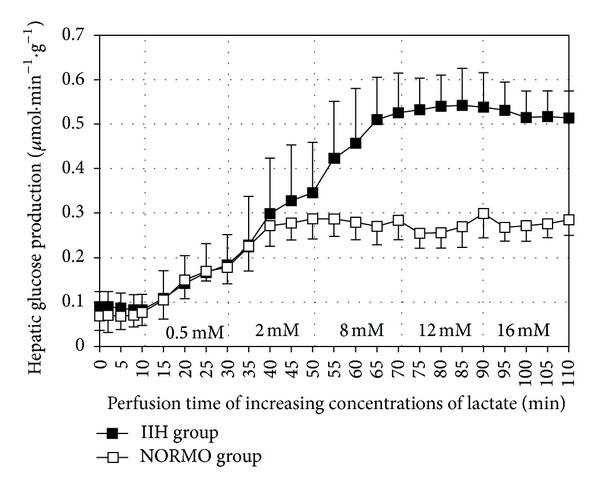
Demonstrative experiments (*n* = 3-4) of glucose production from increasing concentrations of lactate in perfused liver of fasted mice that received intraperitoneal injection of insulin (IIH group, –■–) or saline (NORMO group, –□–). The effluent fluid was collected at 5 min intervals and analyzed for glucose. The areas under curve (the increment of glucose production between 10 and 110 min) of NORMO and IIH group were 39.51 ± 0.19 and 24.96 ± 0.09, respectively.

Figure 1 summarizes a demonstrative experiments and the calculation of the area under curves. Thus, after a preinfusion period of 10 min (basal glucose production), increasing concentrations of lactate were dissolved in the perfusion fluid and infused from 10 to 110 min. The samples of the effluent fluid were collected at 5 min intervals, and the concentration of glucose was measured. The difference in the liver glucose production (LGP) during the infusion period of each concentration of lactate and the basal LGP was used to calculate the AUCs, expressed as *μ*mol·g^−1^. The AUCs presented in Figure 4 and Table 1 were obtained from similar experiments.

### 2.5. Statistical Procedure

The results were expressed as mean ± standard deviation (SD) of 6–8 experiments. The level of significance adopted was 5% (*P* < 0.05). The means were compared through unpaired Student's *t-*test or ANOVA. The calculations and statistical analyses were carried out using GraphPad Prism version 5.0.

## 3. Results

### 3.1. Determination of the Glycemic Curve

**Figure 2:**
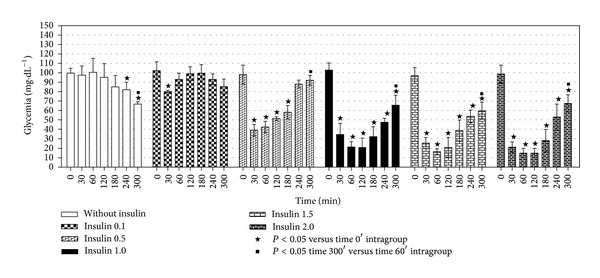
Glycemic response as a function of dose and time after intraperitoneal injection of regular insulin in 14 hr fasted mice. In these experiments saline or increasing doses of insulin (0.1, 0.5, 1.0, 1.5, and 2.0 U·kg^−1^) were injected. Glycemia was measured (mg·dL^−1^) 0, 30, 60, 120, 180, 240, and 300 min after insulin injection. Bars are mean ± standard deviation of 6–8 experiments. ^★^ *P* < 0.05 versus 0 min; ^■^ *P* < 0.05 300 min versus 60 min.

The group that received saline showed decreased glycemia (*P* < 0.05) between the initial time (0 min) and 240 or 300 min after saline injection. Moreover, we observed decrease (*P* < 0.05) of glycemia between 60 and 300 min after saline injection (Figure 2).

Insulin administration (0.1 U·kg^−1^) decreases glycemia (*P* < 0.05) between 0 min and 30 min (Figure 2). Insulin administration (0.5 U·kg^−1^) also promotes the decrease (*P* < 0.05) of the glycemia when 0 min was compared to 30, 60, 120, and 180 min. In addition, an increase (*P* < 0.05) of the glycemia between 60 min and 300 min after insulin injection was observed (Figure 2).

When the doses of 1.0, 1.5, and 2.0 U·kg^−1^ of insulin were evaluated, we observed a fall (*P* < 0.05) in the glycemia from 30 min until 120 min. Additionally, the difference (*P* < 0.05) between 60 min and 300 min for these three doses demonstrated glycemia recovery (Figure 2).

Considering that at the dose of 1.5 U·kg^−1^ or 2.0 U·kg^−1^ all animals had convulsions and half of them died, the dose of 1.0 U·kg^−1^ of regular insulin was used in all experiments.

### 3.2. Evaluation of the Glycemia after the Oral Administration of Saline, Glucose, and Gluconeogenic Precursors in Mice Subjected to IIH

**Figure 3:**
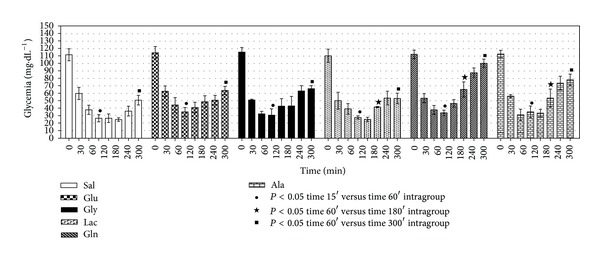
Glycemic response after oral administration of saline (Sal), glucose (Glu), glycerol (Gly), lactate (Lac), glutamine (Gln), or alanine (Ala) during insulin-induced hypoglycemia (IIH). IIH was induced in 14 hr fasted mice with an intraperitoneal injection of regular insulin (1.0 U·kg^−1^). Glycemia (mg·dL^−1^) was determined 0, 15, 30, 60, 120, 180, 240, and 300 min after insulin injection. The oral administration of Sal, Glu (100 mg·kg^−1^), Gly (100 mg·kg^−1^), Lac (100 mg·Kg^−1^), Gln (100 mg·kg^−1^), and Ala (100 mg·kg^−1^) was done 15 min after insulin injection. The bars express the mean ± standard deviation of 6–8 experiments. ^∙^ *P* < 0.05 15 min versus 60 min, ^★^ *P* < 0.05 60 min versus 180 min, and ^■^ *P* < 0.05 60 min versus 300 min.

The effects of the oral administration of saline, glucose, or gluconeogenic precursors in the rats that received 1.0 U·kg^−1^ of regular insulin were compared. In all groups there was a decrease (*P* < 0.05) of the glycemia from 15 min to 60 min, confirming the development of IIH. In addition, all groups showed glycemia recovery (*P* < 0.05). The best performance in terms of glycemia recovery was obtained with glutamine (Figure 3).

Because glycemia recovery started at 180 min after insulin administration, this time was chosen to evaluate liver gluconeogenesis. 

### 3.3. Liver Glucose Production (LGP) from Increasing Levels of Glycerol, Lactate, Alanine, or Glutamine

**Figure 4:**
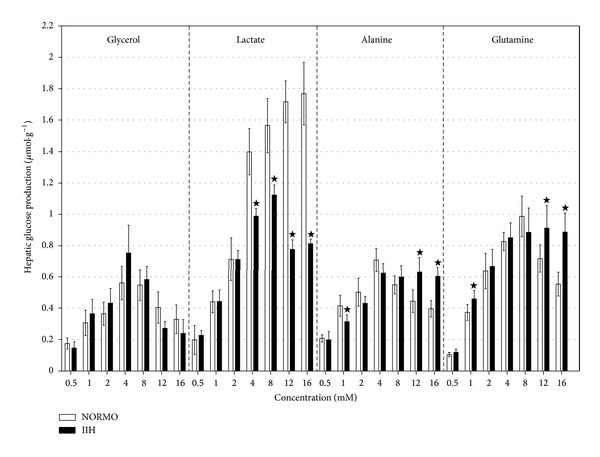
Hepatic production of glucose as a function of increasing concentrations of glycerol, lactate, alanine, and glutamine in 14 hr fasted mice. The animals were subjected to *in situ* liver perfusion 180 min after an intraperitoneal injection of regular insulin (1.0 U·kg^−1^, IIH group) or saline (NORMO group). The bars express the values of the area under curve (*μ*mol·g^−1^), that is, (the increment of glucose production between 10 and 110 min) as mean ± standard deviation of 6–8 experiments. ^★^ *P* < 0.05 IIH versus NORMO.

The infusion of glycerol, lactate, alanine, and glutamine promoted intensification of LGP in all groups (Figure 4). 

 The intensification of LGP from glycerol reached its maximum values at the concentration of 4 mM in both groups. However, at concentrations higher than 4 mM, there was a progressive return of the LGP to the values observed before glycerol infusion (Figure 4). Additionally, from the concentration of 4 mM, the intensification of LGP from lactate was less intense (*P* < 0.05) in the IIH group (Figure 4). 

The intensification of LGP from alanine reaches higher values (*P* < 0.05) in the IIH group at the concentrations of 12.0 and 16.0 mM (Figure 4). Furthermore, the intensification of LGP from glutamine reaches higher values (*P* < 0.05) in the IIH group at the concentrations of 1.0, 12.0, and 16.0 mM (Figure 4). 

### 3.4. Liver Production of Pyruvate from Increasing Levels of Lactate and Liver Production of Pyruvate or Lactate from Increasing Levels of Alanine

The production of pyruvate from increasing levels of lactate showed no significant difference (Table 1). On the other hand, pyruvate and lactate production from increasing levels of alanine was higher (*P* < 0.05) than that of the IIH group at the concentrations of 12.0 and 16.0 mM (Table 1).

### 3.5. Liver Production of Urea from Increasing Levels of Glutamine or Alanine

Similar (IIH versus NORMO) liver urea production from increasing concentrations (from 0.5 to 16.0 mM) of glutamine (AUC ranged from 0.6 to 10.2 *μ*mol·g^−1^) or alanine (AUC ranged from 0.4 to 6.6 *μ*mol·g^−1^) was observed (results not shown). 

## 4. Discussion

Insulin has a potent anabolic effect decreasing the availability of the hepatic glucose precursors [23]. On the other hand, the condition of hyperinsulinemia associated with hypoglycemia leads to the activation of the counterregulatory system with intensification of the release of glucagon and epinephrine [24]. In addition, if hypoglycemia persists for more than two hours, there is an increase in the blood concentration of cortisol and growth hormone and the combination of glucagon, epinephrine, cortisol, and growth hormone could overcome the inhibitory effect of insulin on LGP [7, 25]. Therefore, even with the inhibitory effect of insulin on gluconeogenesis, the activation of the counterregulatory system allows the liver to produce glucose from noncarbohydrate substrates. However, how these mechanisms work in mice, particularly the LGP and glycemia recovery promoted by the administration of gluconeogenic substrates, should be investigated. 

The decreased glycemia (Figure 2) after insulin injection was more intense and prolonged if compared with previous studies in rats [8–12]. Moreover, it was observed that lactate and glycerol were less effective in promoting glycemia recovery compared with the amino acids glutamine and alanine (Figure 3).

Interestingly, oral glutamine promoted better glycemia recovery than glucose (Figure 3), the main antidote used in hypoglycemia [26]. 

In contrast with rats [8, 10, 27], oral glutamine showed better glycemia recovery compared with alanine (Figure 3). This difference could be attributed to the possibility that in mice the catabolism of glutamine in the enterocytes is lower than in rats [28–30].

Since the glycemia recovery depends on LGP, the contribution of glycerol, lactate, alanine, and glutamine to the gluconeogenic activity in livers from hypoglycemic mice was investigated. The choice of these substances was based on the following facts: (1) alanine and glutamine are the most important gluconeogenic amino acid and the most abundant blood amino acid, respectively; (2) lactate and glycerol represent the major final metabolic products of muscle and adipose tissue, respectively; (3) they enter at different points of the gluconeogenesis giving the possibility of an evaluation of specific steps of this metabolic pathway. 

In general, livers from the IIH group showed similar and lower LGP from glycerol and lactate, respectively, while higher (*P* < 0.05) LGP from alanine and glutamine was observed (Figure 4). Therefore, these experiments in the isolated liver help understand the best performance of the amino acids on the glycemia recovery (Figure 3).

The similar LGP from glycerol could be attributed to the fact that glycerol enters the gluconeogenic pathway after the step catalyzed by PEPCK [31, 32]. 

However, the step of entrance in the gluconeogenesis does not explain the liver response to lactate and alanine. Since lactate and alanine are converted to pyruvate, they should have a similar performance in terms of LGP. However, the LGP from alanine and lactate (Figure 4) in livers from IIH mouse was higher (*P* < 0.05) and lower (*P* < 0.05), respectively. These differences could be explained, partly at least, by the fact that the catabolism of lactate, deduced from pyruvate production (Table 1), was similar (NORMO *versus* IIH), while the catabolism of alanine, deduced from pyruvate, and lactate (Table 1) was higher (*P* < 0.05) in the IIH group.

Glutamine, which enters the gluconeogenic pathway at a more distal step compared with alanine or lactate, also showed higher (*P* < 0.05) LGP at the concentrations of 1.0, 12.0, and 16.0 mM (Figure 4) in the IIH group. However, the catabolism of glutamine inferred from urea production did not help explain the higher LGP from glutamine in livers of IIH mice.

Taken together, the results demonstrated that, despite insulin inhibiting gluconeogenesis, an increased gluconeogenic capacity from alanine and glutamine and maintained gluconeogenic capacity from glycerol and lactate were observed. These results are probably due to the fact that long-term IIH (180 min after insulin injection) triggers a counterregulatory response that maintains or even intensifies hepatic gluconeogenesis in mice, an animal model with high sensitivity to the hypoglycemic effect of insulin [33–35].

## 5. Conclusions

Our results demonstrate the superiority of oral glutamine to promote glycemia recovery in comparison with oral glucose opening perspectives for clinical studies to investigate the impact of oral administration of this amino acid during IIH.

## Figures and Tables

**Table 1:** Liver production of pyruvate from increasing concentrations (mM) of lactate and liver production of pyruvate or lactate from increasing concentrations (mM) of alanine in fasted mice. The animals were subjected to *in situ* liver perfusion 180 min after an intraperitoneal injection of regular insulin (1.0 U·kg^−1^, IIH group) or saline (NORMO group). The areas under curve (AUCs), that is, the increment of pyruvate and L-lactate production between 10 and 110 min were calculated as described in Section 2.

Glucose precursor	AUC of liver production of pyruvate and lactate (*μ*mol·g^−1^)			
	Pyruvate		Lactate group HII	
	NORMO group	IIH group	NORMO group	IIH group
Lactate mM				
0.5	0.04 ± 0.01	0.04 ± 0.01	Nd	Nd
1.0	0.04 ± 0.01	0.05 ± 0.02	Nd	Nd
2.0	0.13 ± 0.02	0.18 ± 0.06	Nd	Nd
4.0	0.34 ± 0.08	0.31 ± 0.06	Nd	Nd
8.0	0.74 ± 0.08	0.85 ± 0.09	Nd	Nd
12.0	1.23 ± 0.13	1.25 ± 0.14	Nd	Nd
16.0	1.58 ± 0.15	1.53 ± 0.06	Nd	Nd
Alanine mM				
0.5	0.02 ± 0.01	0.02 ± 0.01	0.05 ± 0.04	0.07 ± 0.02
1.0	0.03 ± 0.01	0.02 ± 0.01	0.14 ± 0.04	0.11 ± 0.03
2.0	0.07 ± 0.02	0.06 ± 0.02	0.23 ± 0.05	0.18 ± 0.05
4.0	0.12 ± 0.04	0.07 ± 0.01	0.44 ± 0.04	0.28 ± 0.06
8.0	0.33 ± 0.10	0.48 ± 0.13	0.60 ± 0.05	0.42 ± 0.02
12.0	0.26 ± 0.08	0.85 ± 0.14*	0.65 ± 0.08	0.84 ± 0.05*
16.0	0.18 ± 0.09	1.16 ± 0.09*	0.63 ± 0.06	1.16 ± 0.11*

Values of the area under curve (*μ*mol·g^−1^) are represented as mean ± standard deviation of 6–8 perfusion experiments. *P < 0.05 NORMO versus IIH. Nd: not determined.
